# Anatomical insights into a five-bellied gastrocnemius muscle: a case report

**DOI:** 10.1007/s00276-026-03832-y

**Published:** 2026-03-06

**Authors:** Wigínio Gabriel Lira-Bandeira, Ivis de Carvalho Medeiros, Mauro Bezerra Montello, Ingrid C. Landfald, George Triantafyllou, Łukasz Olewnik, Judney Cley Cavalcante, Bento João Abreu

**Affiliations:** 1 Group of Study and Research in Human Anatomy (NEPAH), Laboratory of Human Anatomy, Department of Morphology, Federal University of Rio Grande do Norte, Natal, Brazil; 2 Department of Clinical Anatomy, Masovian Academy in Płock, Płock, Poland; 3 Department of Anatomy, School of Medicine, Faculty of Health Sciences, National and Kapodistrian University of Athens, Athens, Greece; 4 VARIANTIS Research Laboratory, Department of Clinical Anatomy, Masovian Academy in Płock, Płock, Poland

**Keywords:** Anatomy, Anatomical variations, Cadaver, Lower limb

## Abstract

The gastrocnemius (GM), a superficial posterior leg muscle, consists of medial and lateral heads and drives ankle plantarflexion and knee flexion. The morphological variability of this muscle has been extensively studied, especially the presence of a third head. Here, a novel five-headed configuration of the GM was identified during the cadaveric dissection of a 30-year-old male. The right GM exhibited five distinct heads, each with independent vascular and neural supply, whereas the contralateral limb displayed a three-headed configuration including a gastrocnemius tertius (GT). Detailed morphometric assessment was performed, and the developmental proximal attachment of the supernumerary heads and its clinical implications were discussed. Although often asymptomatic, these variants can alter biomechanics, narrow the popliteal corridor, and increase the risk of vascular or musculotendinous injury and misdiagnosis. Recognition of complex GM anatomy is therefore essential for surgeons and radiologists. Thus, this case highlights the developmental variability of the posterior leg compartment and underscores the importance of recognizing rare GM variants to ensure accurate diagnosis and safe clinical management.

## Introduction

The gastrocnemius muscle (GM), one of the largest and most superficial muscles in the posterior leg, consists of a medial head (MH) and a lateral head (LH). It plays a key role in ankle plantarflexion and knee flexion, and both heads are innervated by branches of the tibial nerve (S1–S2) and supplied by the sural branches of the popliteal artery. Together with the soleus (SM), the two heads form the thick calcaneal tendon, which has its distal attachment on the medial surface of the calcaneal tuberosity [11]. The literature indicates that the GM exhibits few anatomical variations, most commonly the presence of a third head, known as the gastrocnemius tertius (GT) [1, 20, 21]. The GT may arise from the popliteal surface of the femur, the lateral epicondyle, the knee joint capsule, or even from the long head of the biceps femoris, and it most frequently merges with the MH. Reported prevalence rates vary, with pooled estimates ranging from 4.34 [8] to 10.96% [20]. Other variations in this region have also been described [13, 14], some of which may influence muscle function or even complicate clinical and surgical procedures. In the present work, we describe a previously unreported five-headed variation of the GM and analyze its potential functional and clinical significance.

## Case report

During the routine dissection of a formalin-fixed adult male cadaver (30 years old) at the Human Anatomy Laboratory of the Federal University of Rio Grande do Norte, a five-headed GM was identified in the right lower limb. The heads were numbered from medial (MH) to lateral (fifth head).

The GM was exposed by removing the skin and superficial fascia of the leg. A careful dissection of the popliteal fossa was also performed to assess the innervation of the GM heads. The morphometric characteristics of the five GM heads were measured using a digital caliper, including total length, muscle width, muscle thickness, proximal tendon length, proximal tendon width, and proximal tendon thickness. All measurements were taken three times by the researcher, and the morphometric data are summarized in Table 1.

**Table 1:** Multi-bellied gastrocnemius measurements (30-year-old male, right limb)

Item	Description	Tot len (mm)	MW (mm)	MT (mm)	TL (mm)	TW (mm)	TT (mm)
1	MH	226.58	81.75	18.99	63.36	18.34	5.64
2	LH	217.89	30.11	10.89	49.22	7.48	2.10
3	GT	98.89	15.87	6.93	22.19	3.42	0.29
4	Fourth head	180.26	13.68	16.69	38.56	18.72	2.15
5	Fifth head	69.15	99.71	8.39	34.23	2.11	18.24

MH, medial head; LH, lateral head; GT, gastrocnemius tertius; Tot len, total length; MD, muscle width (middle third); MT, muscle thickness (middle third); TL, proximal tendon length; TW, proximal tendon width; TT, proximal tendon thickness. Heads are enumerated from medial (MH) to lateral (fifth head).

Widths are mediolateral; thicknesses are anteroposterior.

**Fig. 1:**
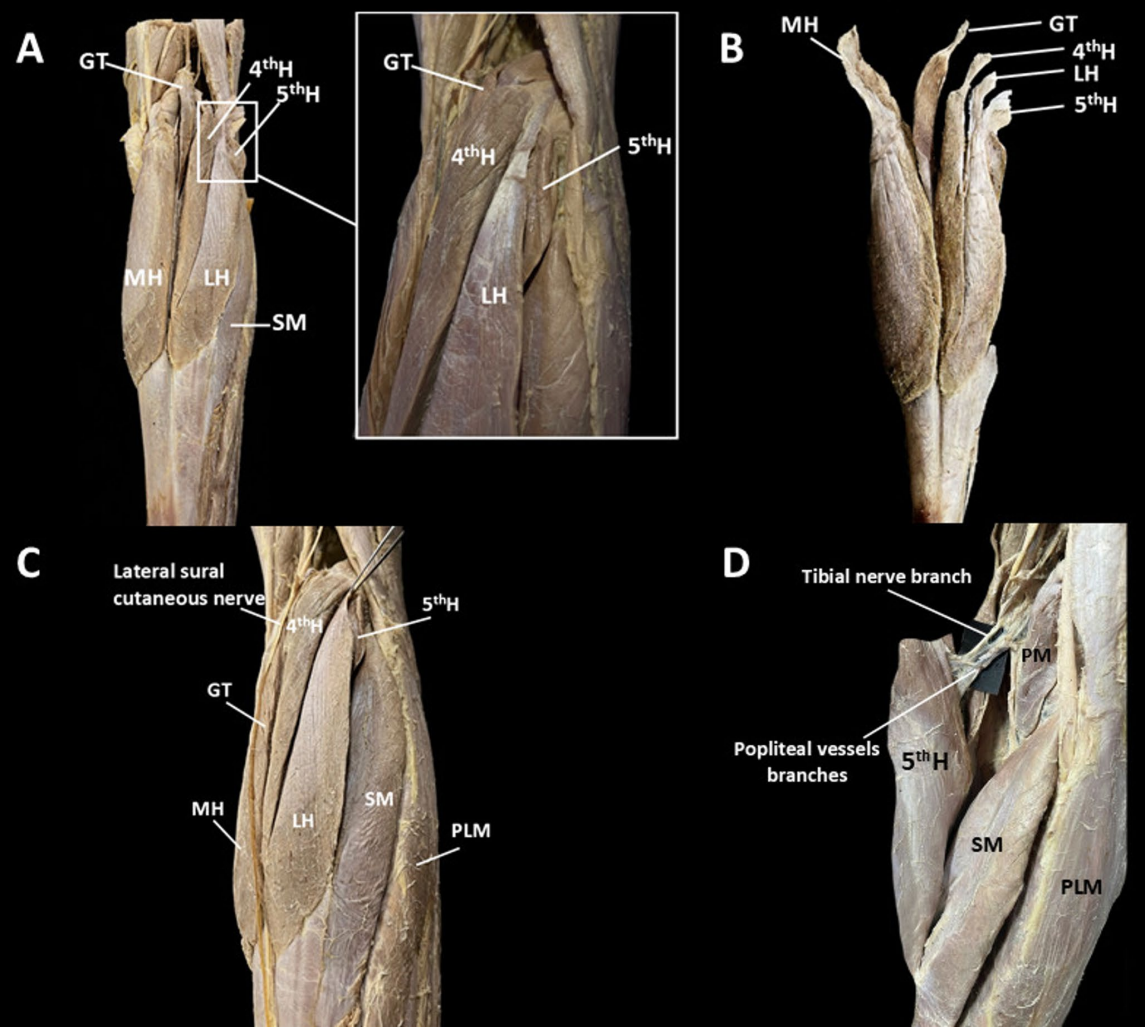
Posterior view of the leg limbs highlighting the right gastrocnemius muscle (GM). **A** Posterior view of the right leg showing the five heads of the GM. **B** Dissected GM showing the distinct proximal attachments of its five heads. **C** Posterolateral view of the five-headed GM showing the lateral sural cutaneous nerve. **D** Posterior view of the fifth head and its neurovascular bundle. CT, calcaneal tendon; GT, gastrocnemius tertius; L, lateral head; MH, medial head; PLM, peroneus longus muscle; PM, plantaris muscle; SM, soleus muscle

In the right lower limb (Fig. 1A, B), the MH was identified with an independent proximal attachment to the posterior supracondylar region of the medial femoral condyle and the popliteal surface of the femoral shaft, just above the knee joint line, where it merged with the articular capsule. This head had its own vascular supply and was innervated by a branch of the tibial nerve.

Laterally, a GT was present, arising independently above the PM and merging with the MH through its own distal tendon (36.22 mm in length, 1.1 mm in width, and 0.09 mm in thickness). The GT was located lateral to the tibial nerve, which issued a delicate branch for its innervation before coursing deeply to supply the remaining heads (Fig. 1A–C).

Between the PM and the head of the fibula, a common tendinous structure was identified, giving rise to the fourth head, the LH, and the fifth head (Fig. 1A, B). Proximally, this tendon had its attachment to the posterior supracondylar region of the lateral femoral condyle, also above the knee joint line, where it fused with the articular capsule. From this elongated proximal attachment, a denser and more prominent tendinous band can be observed, serving as the proximal attachment of the LH. Superiorly, an almost laminar, fan- or funnel-shaped tendinous expansion gives rise to the proximal attachment of the fourth head. Inferior to these structures, a shorter and flattened tendinous portion, arranged in a broader fan-shaped configuration, provides the proximal attachment of the fifth head. The fifth head appeared small on the posterior surface of the leg but was broad, covering the posterior aspects of the third head and the LH, with which it fused distally (Fig. 1D). Fourth and fifth heads joined the LH, converging distally to form the calcaneal tendon together with the MH of the GM. The calcaneal tendon measured 75.56 mm in length, 13.45 mm in width, and 9.07 mm in thickness.

Interestingly, a three-headed configuration of the GM was observed in the contralateral limb, with a GT arising from the tendon of the lateral head and merging into the distal third of the lateral head, resembling the fourth head identified in the right limb.

## Discussion

In this report, a novel five-headed configuration of the right GM is described, whereas the contralateral limb displayed a GT functioning as a “third head”. Beyond the presence of the GT, other variations of the GM have been reported, including the occasional absence of the lateral head or even the entire muscle [17], absence of the calcaneal tendon with the MH and LH inserting directly into the bone [15], aberrant origins from the thigh muscles [16], and additional supernumerary heads [2, 13]. Classical anatomical literature reports the presence of a third head of the GM, commonly referred to as the GT [3], and occasionally describes a bifid configuration of this additional belly [10]. Moreover, the literature also documents persistent separation of the medial and lateral heads up to their distal attachment to the calcaneal tendon or complete dissociation from the soleus muscle, cases of hypoplasia or absence of one head, the presence of sesamoid ossicles (e.g., the fabella) within the proximal tendinous attachments, and anomalous muscular slips that fuse the heads into a single muscular mass [18]. Further reported variations include aberrant connections with adjacent muscles such as the plantaris, soleus, and popliteus, as well as “ischio-calcaneal” bundles extending from the hamstrings to the calcaneal tendon [18].

In contrast to the findings of Rodrigues et al. [13], which described a GM in which each main head (LH and MH) was attached to the femur by three symmetrical and robust heads, the configuration observed in the present case is entirely atypical, highlighting an unusual pattern of developmental variation in the superficial muscles of the posterior leg compartment. It is well-established that the posterior leg compartment is poorly differentiated before the second month of development, and variations in these muscles likely result from incomplete separation of the original anlage into three or more distinct muscles. In this context, the GT and the fourth and fifth heads may be considered congenital muscle or tendinous structures originating from different regions of the distal dorsal surface of the femur. These supernumerary heads typically merge with the LH, MH, or both heads of the GM [20].

The unusual configuration described here likely amplifies the clinical risks already associated with supernumerary heads in the popliteal fossa, which may include leg pain, tenderness in the popliteal region, and reduced arterial pulsations in the leg and foot [12, 23]. Indeed, accessory bellies are not rare on MRI, most commonly presenting as a “third head” coursing lateral to the popliteal vessels in approximately 2% of routine knee examinations. Although frequently asymptomatic, their additional bulk and atypical trajectories may narrow the popliteal corridor and modify local shear and strain dynamics [9]. Within this anatomical context, the spectrum of popliteal artery entrapment syndrome (PAES) offers a coherent pathophysiological framework: both anatomic (Rich types I–V) and functional forms can be precipitated or exacerbated by anomalous or hypertrophic gastrocnemius slips, and dynamic imaging with duplex and CTA/MRA during provocative maneuvers is recommended to unmask compression that appears occult in neutral [5]. Beyond arterial disease, venous compromise warrants consideration: isolated popliteal vein entrapment from an accessory slip of the lateral gastrocnemius has been reported in young, athletic patients and can present with deep vein thrombosis (DVT)-like swelling or true thrombosis, with symptom relief after resection of the anomalous muscle [22]. Symptom overlap with chronic exertional compartment syndrome (CECS) is substantial; misdiagnosis in either direction is well documented, and coexistence (functional PAES ± CECS) is increasingly recognized, supporting a dual diagnostic pathway in refractory exertional calf pain [7, 8]. Complex multi-belly anatomy may also heighten the likelihood of musculotendinous-junction injury (“tennis leg”) and intramuscular haematoma, entities that commonly mimic DVT; a structured algorithm (clinical exam → duplex to exclude DVT → targeted US/MRI) helps avoid unnecessary anticoagulation and guides rehabilitation [4]. From a surgical standpoint, supernumerary bellies can obscure or displace the tibial nerve and popliteal vessels during posterior knee approaches and posterior cruciate ligament (PCL) reconstruction; pre-operative MRI mapping and meticulous protection are essential given the millimetric artery-to-PCL distances under arthroscopic conditions [6].

In addition, because MH flaps are a workhorse for peri-knee coverage, recognizing a multi-bellied configuration pre-harvest may influence side selection, arc-of-rotation planning, and pedicle handling to avoid iatrogenic neurovascular traction or partial devascularization of an accessory head [19]. Collectively, these considerations argue for (i) vigilant dynamic imaging in exertional symptoms, (ii) routine description of variant bellies and their relation to vessels/nerve in radiology reports, and (iii) explicit surgical planning notes whenever posterior approaches, vascular decompressions, or gastrocnemius-based reconstructions are contemplated [5, 9].

Finally, clear documentation and high-quality visualization of anatomical variants are essential components of modern anatomical practice. Beyond cataloging variations, detailed dissections and accurate imaging provide a reliable foundation for anatomical education, surgical planning, and clinical interpretation. In this context, the present report contributes to the growing need for well-documented anatomical evidence of morphological diversity.

## Conclusion

In summary, the reported five-headed GM underscores the developmental variability of the posterior leg compartment. While often asymptomatic, such variants can significantly affect biomechanics, constrict the popliteal corridor, and increase the risk of vascular entrapment, musculotendinous injury, or misdiagnosis as DVT or CECS.

Awareness and careful recognition of these variations are therefore critical for surgeons, radiologists, and other clinicians to ensure accurate diagnosis and safe management. In this sense, dynamic studies detailed radiological reporting, and preoperative mapping should be standard when variant GM slips are suspected, ensuring better outcomes in vascular, orthopedic, and reconstructive procedures.

## Data Availability

No datasets were generated or analyzed during the current study.
